# A low dose lipid infusion is sufficient to induce insulin resistance and a pro-inflammatory response in human subjects

**DOI:** 10.1371/journal.pone.0195810

**Published:** 2018-04-12

**Authors:** Hanyu Liang, Helen Lum, Andrea Alvarez, Jose de Jesus Garduno-Garcia, Benjamin J. Daniel, Nicolas Musi

**Affiliations:** 1 Barshop Institute of Longevity and Aging Studies, UT Health San Antonio, San Antonio, Texas, United States of America; 2 Department of Medicine-Diabetes Division, UT Health San Antonio, San Antonio, Texas, United States of America; 3 Geriatric Research Education and Clinical Center, Audie L. Murphy VA Medical Center, San Antonio, Texas, United States of America; 4 Department of Microbiology, UT Health San Antonio, San Antonio, Texas, United States of America; Medical University of Vienna, AUSTRIA

## Abstract

**Objective:**

The root cause behind the low-grade inflammatory state seen in insulin resistant (obesity and type 2 diabetes) states is unclear. Insulin resistant subjects have elevations in plasma free fatty acids (FFA), which are ligands for the pro-inflammatory toll-like receptor (TLR)4 pathway. We tested the hypothesis that an experimental elevation in plasma FFA (within physiological levels) in lean individuals would upregulate TLR4 and activate downstream pathways (e.g., MAPK) in circulating monocytes.

**Research design and methods:**

Twelve lean, normal glucose-tolerant subjects received a low dose (30 ml/h) 48 h lipid or saline infusion on two different occasions. Monocyte TLR4 protein level, MAPK phosphorylation, and expression of genes in the TLR pathway were determined before and after each infusion.

**Results:**

The lipid infusion significantly increased monocyte TLR4 protein and phosphorylation of JNK and p38 MAPK. Lipid-mediated increases in TLR4 and p38 phosphorylation directly correlated with reduced peripheral insulin sensitivity (M value). Lipid increased levels of multiple genes linked to inflammation, including several TLRs, CD180, MAP3K7, and CXCL10. Monocytes exposed *in vivo* to lipid infusion exhibited enhanced *in vitro* basal and LPS-stimulated IL-1β secretion.

**Conclusions:**

In lean subjects, a small increase in plasma FFA (as seen in insulin resistant subjects) is sufficient to upregulate TLR4 and stimulate inflammatory pathways (MAPK) in monocytes. Moreover, lipids prime monocytes to endotoxin. We provide proof-of-concept data in humans indicating that the low-grade inflammatory state characteristic of obesity and type 2 diabetes could be caused (at least partially) by pro-inflammatory monocytes activated by excess lipids present in these individuals.

## Introduction

Obesity-related chronic low-grade inflammation is an important element in the pathogenesis of insulin resistance and type 2 diabetes. Manifestations of inflammation include increased levels of circulating cytokines [1], activation and recruitment of immune cells to insulin-responsive tissues (especially adipose [2]), and stimulation of pro-inflammatory signaling pathways [3] in various cell types. Accumulating research findings suggest that monocytes play a key role in the development of insulin resistance. Circulating monocytes are activated in obesity [4] and type 2 diabetes [5]. These pro-inflammatory monocytes infiltrate adipose tissue [2], where they become tissue macrophages and secrete chemokines to further facilitate immune cells recruitment, thus setting up a positive feed-forward loop that potentiates inflammation. Monocytes and macrophages are among the major sources of cytokines such as tumor necrosis factor (TNF)α and interleukin (IL)-1β. These inflammatory mediators inhibit insulin signaling through various mechanisms including activating mitogen-activated protein kinase (MAPK) [6] and inhibitor of κB kinase β (IKKβ)-nuclear factor κB (NFκB) [3]. Despite the vast mechanistic evidence linking inflammation and insulin resistance, the root cause behind the low-grade inflammatory state seen in obesity and type 2 diabetes remains unclear, particularly in humans.

TLR4 is a pattern recognition receptor highly enriched in monocytes. It mediates lipopolysaccharide (LPS)-induced inflammatory response [7] and therefore is critical for activation of innate immunity. Fatty acids acylated in lipid A moiety of LPS are essential for the activation of TLR4. Moreover, fatty acids alone are potent activators of TLR4 [8]. Given that insulin-resistant subjects (either with obesity or type 2 diabetes) have elevated plasma FFA concentrations due to excessive lipolysis [9], TLR4 and downstream pathways in monocytes could be a mechanistic link between FFA and inflammation. Consistent with this notion, previously we observed that peripheral blood mononuclear cells isolated from obese and type 2 diabetic subjects have significantly elevated levels of TLR4 protein compared to healthy lean controls [10]. In addition, stearic acid (a highly abundant fatty acid in plasma) increased TLR4 protein levels by 7-fold in primary human mononuclear cells *in vitr*o [10]. Nonetheless, there is limited evidence from human studies demonstrating that changes in FFA levels, within a physiological range, can modulate inflammatory pathways in peripheral blood cells. Here, we tested the hypothesis that an experimental elevation in plasma FFA in lean individuals would upregulate TLR4 and stimulate downstream (e.g., MAPK) pathways in circulating monocytes. If positive, these findings would provide proof-of-concept that mild FFAs elevations can induce a pro-inflammatory state in human subjects.

## Materials and methods

### Study design

We recruited 12 lean healthy subjects through local advertisement. Details of the study design were published elsewhere [11]. Briefly, all subjects were sedentary and had stable body weight (±1 kg) for more than 3 months before entering the study. Each subject underwent a medical history, physical examination, screening laboratory tests, and an oral glucose tolerance test (OGTT) to document normal glucose tolerance. The study was approved by the Institutional Review Board of the University of Texas Health Science Center at San Antonio. All subjects gave informed written consent.

Within 30 days of the OGTT, subjects were admitted to the Bartter Research Unit at the Audie L. Murphy VA Medical Center. Subjects were studied on 2 occasions separated by a washout period of 4–8 weeks. They were infused once with Intralipid® 20% (Baxter Healthcare, Deerfield, IL, USA) and once with saline, in random order. Both Intralipid® and saline were infused intravenously for 48 h at 30 ml/h. During this time, subjects were ambulatory and consumed a weight-maintaining diet consisting of 55% carbohydrate, 25% fat, and 20% protein.

On the third day of admission for saline/lipid infusion, after an overnight fast, subjects underwent a 180 min euglycemic, hyperinsulinemic (80 mU m^−2^ min^−1^) clamp study as described previously [12]. The saline/lipid infusion was administered throughout the clamp. Insulin-stimulated glucose metabolism (M value) was determined as the mean glucose infusion rate during the last 30 min of the clamp [13]. Blood draws for monocyte isolation were performed twice during each admission, once before the saline/lipid infusion on day 1 and once before the insulin clamp on day 3, towards the end of the infusion. Sixty minutes after the blood draw, blood samples were processed for monocyte isolation and flow cytometry analysis.

### LPS assay

LPS concentration was determined using a Limulus Amoebocyte Lysate (LAL) assay kit (Lonza, Walkersville, MD) according to manufacturer’s instruction. Plasma samples were diluted 1:20 in endotoxin free water and heated at 70° C for 10 min prior to the assay to inactivate factors that interfere with LAL assay [14]. Samples were run in duplicate and concentrations extrapolated from a standard curve containing known concentrations of LPS from 0.01 to 1 endotoxin unit (EU)/ml. All materials used were sterile and labeled to be pyrogen-free by the manufacturers. All reagents were furthered tested to verify that they are endotoxin-free.

### Blood monocyte isolation

Monocytes were isolated from peripheral blood mononuclear cells (PBMCs) using a Dynabeads® Untouched™ Human Monocytes Kit (Life Technologies, Grand Island, NY) by negative selection. Briefly, blood was collected using tubes coated with ethylenediaminetetraacetic acid (EDTA) and was diluted 1:1 with phosphate-buffered saline (PBS) supplemented with 2 mM ethylenediaminetetraacetic acid (EDTA). Diluted blood was carefully layered over Histopaque-1077 (Sigma-Aldrich) and centrifuged at 800*g* for 20 min at room temperature. PBMCs were recovered at the interface between plasma and Histopaque-1077 and washed 3 times with ice-cold phosphate buffered saline (PBS) supplemented with 0.1% bovine serum albumin (BSA). PBMCs were then resuspended in an isolation buffer (PBS supplemented with 0.1% BSA and 2 mM EDTA). For further monocyte isolation by negative selection, a cocktail of antibodies towards non-monocytes was added to the suspended PBMCs and allowed to bind to the cells. After a 1 min wash with isolation buffer, magnetic Dynabeads® were added to bind to antibody-labeled cells. The bead-bound cells were then separated on a magnet and discarded. The remaining negatively isolated monocytes were used in downstream applications. Purity of monocytes was evaluated by flow cytometry. More than 95% of the cells were CD14^+^, a marker for monocytes. A portion of the isolated monocytes was plated in a 24-well ultra-low attachment plate (2 x 10^5^ cells in 0.5 ml) and cultured in RPMI 1640 medium at 37°C and 5% CO_2_ in a humidified cell culture incubator, both alone and in the presence of 1 μg/ml LPS [15] for 18 h. The monocyte supernatants were then collected, centrifuged, and stored at -70°C for cytokine quantification by multiplex assay. All reagents used were endotoxin-free.

### Flow cytometry

TLR2 and TLR4 cell surface expression was determined with flow cytometry. Briefly, 150,000 isolated monocytes were washed with FACS buffer (PBS supplemented with 0.5% BSA and 0.09% NaN_3_, pH7.2–7.4) and incubated with an antibody cocktail (anti-TLR4 phycoerythrin [PE]: Ebioscience #12-9917-42, anti-CD14 PE-Cy5.5: Beckman Coulter #A70204, and anti-TLR2 Alexa Fluor [AF] 488: Ebioscience #53-9024-82) in the dark on ice for 30 min. Cells were then washed and resuspended in FACS buffer for flow cytometry analysis.

Intracellular levels of p-p38, p-JNK, and p-ERK in peripheral blood CD14^+^ monocyteswere determined with a Phospho flow cytometry method as described by Chow et al. [16] with some modifications. Briefly, whole blood was collected using EDTA-coated tubes. One hundred μl of blood was left unstimulated or was stimulated with 1 μg LPS for 10 min at 37°C, followed by fixation in 4% formaldehyde for 10 min at room temperature, and subsequent white cell permeabilization and red cell lysis using 0.1% Triton X-100 for 15 min at 37°C. White cells were washed in wash buffer (ice-cold PBS with 4% FBS) and were treated with 50% MeOH for 10 min on ice to unmask phosphor-epitopes. After washing, cells were stained with an antibody cocktail (anti-pERK1/2 AF 488: Cell Signaling#4374, anti-CD14 PE-Cy5.5: Beckman Coulter #A70204, anti-pJNK AF 647: Cell Signaling #4374, anti-CD3 allophycocyanin [APC] AF 750: Beckman Coulter #A66329, anti-pP38 Pacific Blue: BD Biosciences #560313, and anti-CD45 Krome Orange: Beckman Coulter #A96416) in the dark at room temperature for 30 min. Cells were then washed once, resuspended in 300 μl of wash buffer, and analyzed by flow cytometry.

Flow cytometry was performed on a BD LSR II flow cytometer equipped with blue, violet, and red lasers (BD Biosciences, San Jose, CA). Compensation was performed for each color. Digital data were acquired with BD Diva software and 100,000 cells collected per sample. Data were further analyzed using FlowJo software. Monocyte gating strategy is shown in S1 Fig. The levels of receptor cell surface expression and intracellular MAPK phosphorylation in the CD14^+^ monocytes were determined by measuring the median fluorescence intensity (MFI) in the appropriate detector.

### Multiplex assays

IL-1β, IL-1Ra, IL-6, MCP-1 and TNFα concentration in cell culture medium was measured simultaneously with a multiplex assay using a Luminex 200™ device (EMD Millipore, Billerica, MA) according to the manufacturer’s instruction. Briefly, samples were incubated with cytokine capture antibody-coupled magnetic beads overnight in a 96-well plate. Next, beads were washed and incubated with biotinylated detection antibody cocktail. Streptavidin-phycoerythrin was then added. Lastly, beads were washed, resuspended with sheath fluid, and analyzed on a Luminex 200™. Cytokine concentrations were calculated from standard curves constructed with recombinant cytokines of known concentrations.

### Real-time RT-PCR

Total RNA were isolated using TRIzol reagent, and cDNA was generated using a RT^2^ First Strand Kit (Qiagen, Valencia, CA). The expression of 84 genes within TLR regulated signaling pathways was determined using a human TLR Signaling Pathway RT^2^ Profiler PCR Array (Qiagen) according to the manufacturer’s instruction. Gene expression was normalized using a panel of five housekeeping genes.

### Statistical analysis

All data are represented as the mean value of each group ± standard error of mean (SEM). Differences between groups were determined using paired Student's *t*-test or Wilcoxon signed-rank test where appropriate. Spearman correlation was utilized to determine correlation values between variables. Statistical significance was assumed at *P*<0.05. Statistical analyses were done using GraphPad Prism software. As indicated in the figure legends, some samples were missing due to low cellular yield or technical problems with the FACS. Phospho flow assays were not done in two subjects because the assay was being developed in the lab. Outliers, defined as values ˃ 3 standard deviations from the mean, were not included in the data analysis.

## Results

### Subject characteristics

Subjects’ clinical and laboratory values were reported in a previous publication [11]. All 12 subjects (sex: 5M/7F; age: 40 ± 3 yrs; BMI: 23.5 ± 0.7 kg/m^2^; HbA1C: 5.4 ± 0.1%; fasting plasma glucose: 95 ± 2 mg/dl; 2h plasma glucose: 102 ± 5 mg/dl) were lean with normal glucose tolerance.

### Effect of lipid infusion on metabolic parameters

Subjects' metabolic profiles after saline and lipid infusion are shown in S1 Table. Plasma FFA concentration, M value, and HOMA-IR index values were published elsewhere [11]. Saline infusion did not alter FFA levels (374 ± 26 vs. 420 ± 25 μmol/l), whereas lipid infusion increased plasma FFA concentration by 36% (355 ± 33 vs. 484 ± 24 μmol/l, *P*<0.01). This small elevation of FFA led to impaired glucose homeostasis, as shown by lower M value (17%, *P*<0.05) and higher HOMA-IR (60%, *P*<0.05), fasting plasma glucose (7%, *P*<0.05), and fasting plasma insulin (46%, *P*<0.05). There was no difference in plasma LPS concentration between saline and lipid groups (S2 Fig).

### Effects of lipid infusion on TLR cell surface expression and intracellular MAPK activation in peripheral blood monocytes

**Fig 1:**
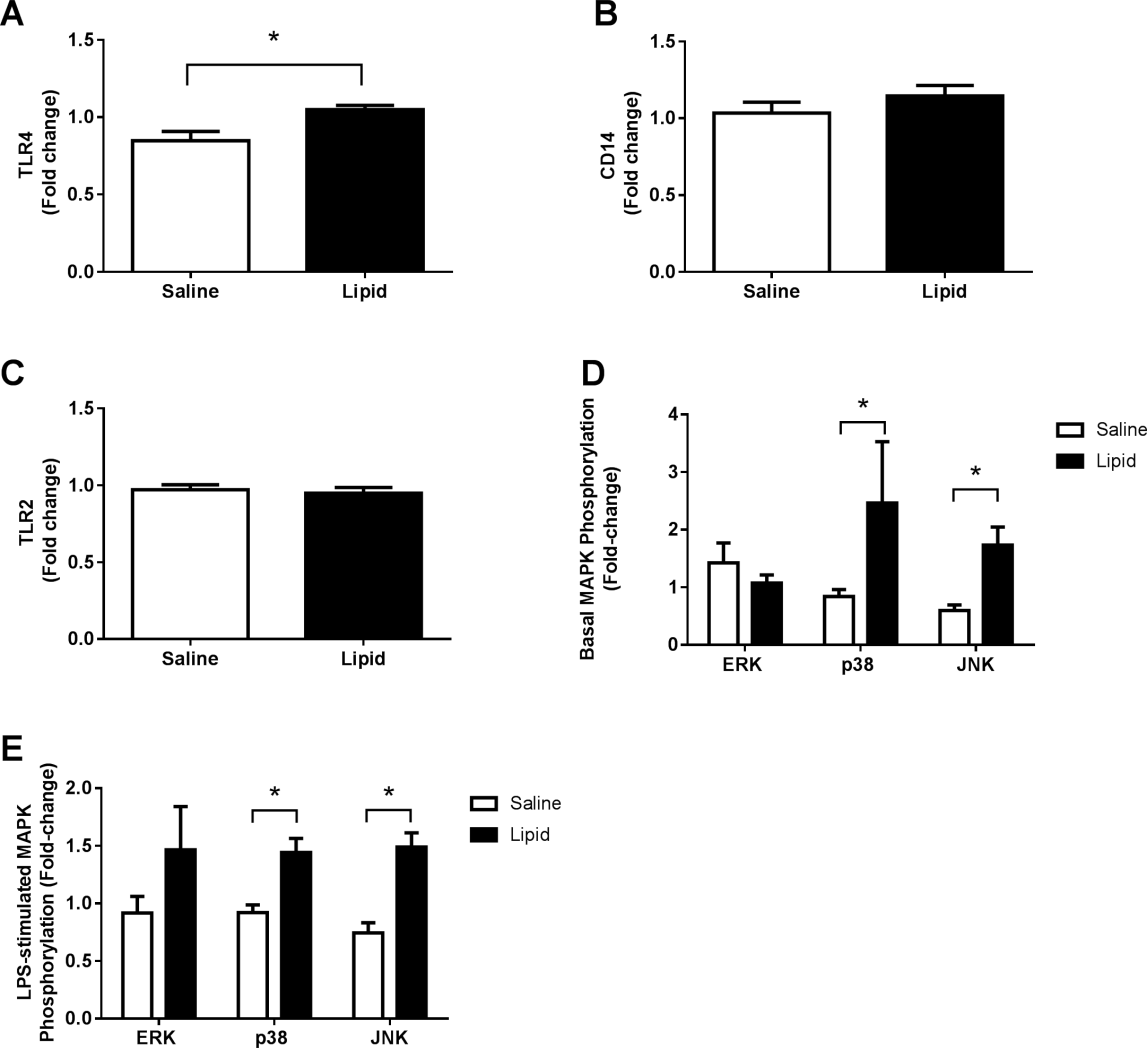
Effect of lipid infusion on TLR cell surface expression and intracellular MAPK phosphorylation in peripheral blood monocytes. Cell surface protein levels of TLR4 (A), CD14 (B) and TLR2 (C) and basal (D) and LPS-stimulated (E) MAPK phosphorylation were determined by flow cytometry as described under ‘Materials and methods’. All values are the mean ± SEM of data obtained from 10–12 subjects. *P<0.05.

Because FFA activates TLR4 *in vitro*, we determined whether 48 h of lipid infusion also would upregulate TLR4 protein in monocytes *in vivo*. Lipid infusion increased TLR4 cell surface expression by 26% compared to saline (*P*<0.05) (Fig 1A). The lipid-induced increase (Δ) in TLR4 level directly correlated with the reduction (Δ) in M (r = 0.59, *P*<0.05). Lipid infusion did not affect CD14 and TLR2 cell surface expression (Fig 1B and 1C).

In addition to increasing TLR4 protein level, the lipid infusion enhanced basal and LPS-stimulated JNK phosphorylation by 2.9- and 2-fold, respectively (Fig 1D and 1E). The lipid infusion also increased p38 MAPK phosphorylation at baseline (2.9-fold) and after LPS stimulation (1.6-fold); increased basal p38 phosphorylation (Δ) directly correlated with reduced M (Δ) caused by lipid infusion (r = 0.93, *P*<0.005). ERK phosphorylation was not affected by lipid or saline (Fig 1D and 1E).

### Effects of lipid infusion on inflammation-related genes

**Fig 2:**
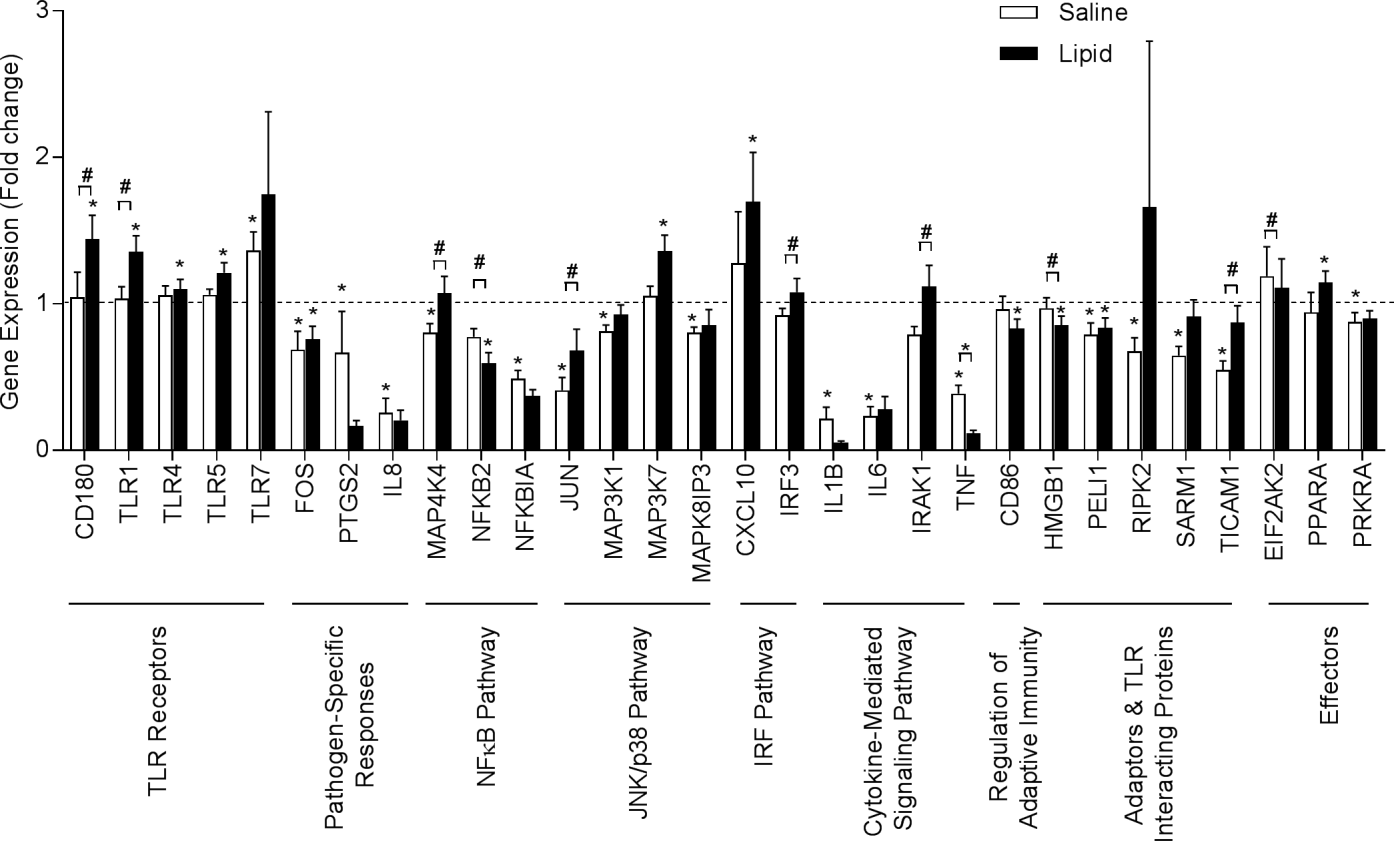
Effects of lipid infusion on expression of genes in the TLR signaling pathway in peripheral blood monocytes. Expression of 84 genes central to TLR-mediated signal transduction was determined using a human TLR Signaling Pathway RT^2^ Profiler PCR Array as described under ‘Materials and methods’. All values are mean ± SEM of data obtained from 12 subjects. *P<0.05 pre- vs. post-infusion within group; #*P*<0.05 post-saline vs. post-lipid.

We profiled expression of 84 genes in the TLR signaling pathway using a TLR PCR array (Fig 2). Compared to baseline, lipid infusion increased mRNA levels of several TLRs including TLR1 (35%), TLR4 (10%), TLR5 (21%), and CD180 molecule (CD180, 44%), a cell surface protein that works in concert with TLR4 to regulate immune responses. Lipid infusion also increased TLR-regulated genes such as mitogen-activated protein kinase kinase kinase 7 (MAP3K7, 36%), a potent activator of JNK, and chemokine (C-X-C motif) ligand 10 (CXCL10, 69%), a chemokine-promoting chemoattractant of immune cells. Lipid infusion caused a mild increase (15%) in mRNA expression of peroxisome proliferator-activated receptor alpha (PPARA), another TLR effector gene.

Comparisons of post-saline vs. post-lipid expression levels revealed increases in toll-like receptor adaptor molecule 1 (TICAM1, 59%). Several genes downstream of the TLRs also were elevated in post-lipid samples (vs. post-saline), including mitogen-activated protein kinase kinase kinase kinase 4 (MAP4K4, 34%) in the NFκB pathway, Jun proto-oncogene (JUN,66%) in the JNK/p38 pathway, interferon regulatory factor 3 (IRF3, 17%) in the IRF pathway, and interleukin-1 receptor-associated kinase 1 (IRAK1, 41%) in cytokine-mediated signaling pathways (Fig 2).

The gene expression level of 15 genes was lower after saline infusion. These genes included pathogen response proteins such as prostaglandin-endoperoxide synthase 2 (PTGS2, 33%) and IL-8 (74%), MAP4K4 (20%) and nuclear factor of kappa light polypeptide gene enhancer in B-cells (p49/100) (NFKB2, 23%) within the NFκB pathway; JUN (59%), mitogen-activated protein kinase kinase kinase 1 (MAP3K1, 19%) and mitogen-activated protein kinase 8 interacting protein 3 (MAPK8IP3, 20%), of the JNK/p38 pathway; IL1B (79%), IL6 (68%), and TNF (76%) in the cytokine-mediated signaling pathway; receptor-interacting serine-threonine kinase 2 (RIPK2, 32%), sterile alpha and TIR motif containing 1 (SARM1, 35%), and TICAM1 (45%) in the adaptor and TLR interacting protein category; and TLR effector such as protein kinase interferon-inducible double stranded RNA dependent activator (PRKRA, 12%). The expression of FOS, and PELI1 was reduced by both saline and lipid. TLR7 expression was elevated after both saline (36%) and lipid (75%) although the effect of lipid did not reach statistical significance.

### Effects of lipid infusion on cytokine production in cultured monocytes

**Fig 3:**
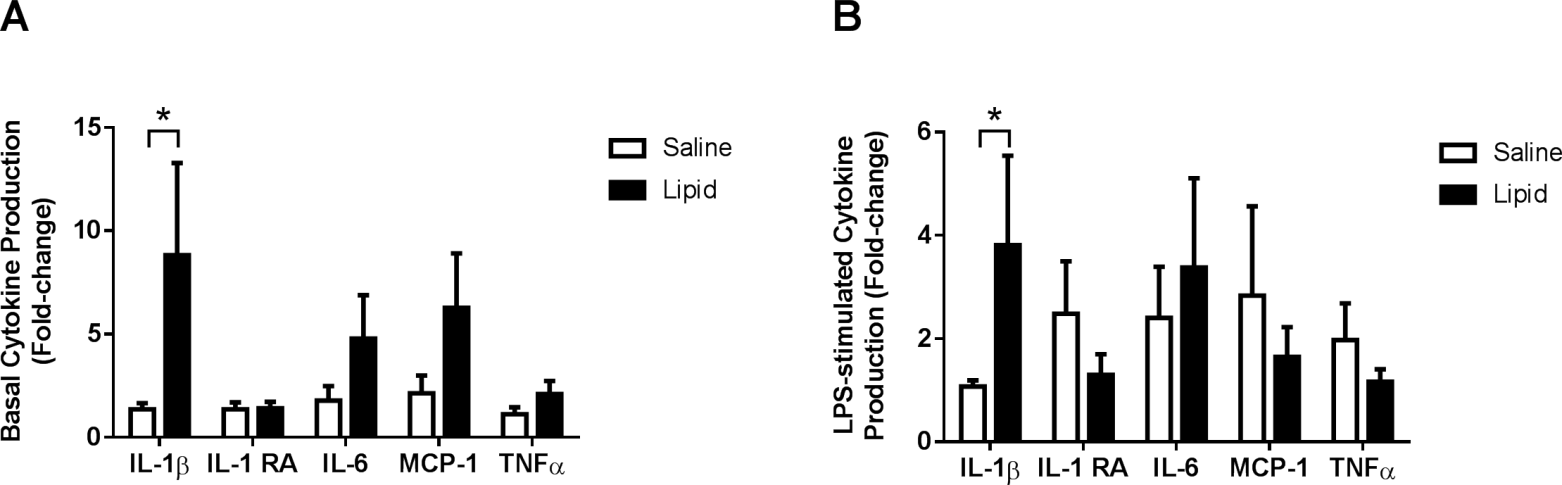
Effects of lipid infusion on cytokine production in cultured monocytes. Monocytes were isolated using a Dynabeads® Untouched™ Human Monocytes Kit. Two hundred thousand cells were either left untreated or treated with 1 μg/ml LPS for 18 h. Basal (A) and LPS-stimulated (B) cytokine production was determined by multiplex assay as described under ‘Materials and methods’. All values are mean ± SEM of data obtained from 12 subjects. *P<0.05.

We assessed whether *in viv*o exposure to lipid affected basal and LPS-stimulated cytokine production by monocytes *in vitro*. Lipid infusion increased basal IL-1β production by 7-fold compared to saline (*P*<0.05) (Fig 3A). There was a trend toward higher monocyte production of IL-6 (2.7-fold, *P* = 0.09), MCP-1(1.8-fold, *P* = 0.12), and TNFα (1.8-fold, *P* = 0.11) at baseline, following lipid infusion. LPS-stimulated IL-1β production (*in vitro*) was 3.4-fold higher (*P*<0.05) in monocytes from subjects exposed *in vivo* to lipid compared to saline. Levels of other cytokines were not different between saline and lipid infusions (Fig 3B).

## Discussion

There is a large body of data derived mainly from *in vitro* and animal studies linking TLR4 to insulin resistance [8, 17–19]. Yet, very few studies have evaluated the role of TLR4 in insulin resistant subjects and the mechanisms underlying altered TLR4 signaling in these individuals. Previously we reported that obese and type 2 diabetic subjects have increased TLR4 expression and signaling in skeletal muscle [20]. In the present study, we used a lipid infusion model to investigate whether FFA activates TLR4 signaling in monocytes in humans. We demonstrate that a low dose lipid infusion is sufficient to upregulate TLR4 mRNA and protein levels in circulating monocytes. Increased expression of TLR4 in monocytes was associated with increased JNK/p38 MAPK phosphorylation/activation and gene expression of TLR-regulated genes. Lipid infusion also greatly increased the ability of monocytes to secrete IL-1β, a potent pro-inflammatory cytokine. These data indicate that mild elevations in FFA levels, as seen in obese and type 2 diabetic subjects, may contribute to the pro-inflammatory state (i.e., upregulation of TLR4 levels and downstream signaling) in circulating monocytes. Furthermore, lipid-induced increases in TLR4 and p38 phosphorylation directly correlated with reduced M value, further suggesting that TLR4 signaling in monocytes could play a role in lipid-induced insulin resistance in humans.

Our findings are in line with those from a previous study that demonstrated that a high-fat, high-carbohydrate meal can increase TLR4 protein levels in human mononuclear cells [21]. Also consistent with our findings, TLR4 expression in peripheral mononuclear cells was reduced in overweight individuals with metabolic syndrome undergoing weight loss [22]. These studies highlight the ability of TLR4 to function as a metabolic/nutritional sensor. One limitation of these studies, however, is that these interventions result in changes in several macronutrients (e.g., carbohydrates) and hormones (e.g., incretins) that can affect inflammatory pathways. The present study allows the independent assessment of lipids on inflammation and insulin resistance *in vivo*.

We measured MAPK phosphorylation as an indicator of TLR4 activation. Nonetheless, MAPKs can be activated via TLR4-dependent and -independent mechanisms. Because of limitations in the amount of blood available from research participants we chose to analyze TLR signaling via FACS and gene expression profiling, rather than immunoprecipitation and Western analysis that are required to assess interactions between TLR4 and its adapter protein, MyD88. On the other hand, FACS eliminates the need for blood cell isolation that can artificially alter signaling cascades. The specificity of TLR pathway activation can be assessed in future studies combining immunoprecipitation-Western blotting, FACS and gene profiling.

The increases in monocyte TLR4 and phosphorylated JNK and p38 levels caused by lipid infusion were associated with enhanced production of multiple cytokines, including IL-1β, IL-6, MCP-1, and TNFα, all of which have been implicated in insulin resistance and type 2 diabetes. It is thought that monocytes/macrophages contribute significantly to the circulating levels of these cytokines in humans. MAPKs activate transcription factors such as c-Jun, ATF-1, ATF-2 and CREB, which in turn promote production of cytokines. In addition, blocking the function of MAPKs diminishes cytokine secretion [23]. Therefore, TLR4-MAPK activation by lipids may contribute to the increases in cytokine concentration typically seen in insulin resistant subjects.

Among the MAPKs that we measured, the increase of p38 phosphorylation after lipid exposure was the greatest and strongly correlated with reduction of muscle insulin sensitivity, highlighting the role of monocyte p38 in whole body inflammation and glucose metabolism. p38 was first discovered as a kinase that regulated LPS-stimulated TNFα and IL-1 production in the monocyte [24]. There are four members of the p38MAPK family: p38α, p38β, p38γ and p38δ, with p38α being the most well characterized and predominant form of p38 in many tissues. The whole body p38α KO is embryonically lethal [25] and cytokine production is reduced when macrophage-specific p38α KO mice are challenged with LPS [26]. p38α regulates cytokine biosynthesis at the transcriptional and translational levels [27]. The IL-1β, IL-6, MCP-1 and TNFα genes all share an AU-rich element (ARE) at their 3'-untranslated region to which p38α binds. The presence of this element shortens the half-lives of the mRNA containing them, or blocks their translation. In the present study, lipid-exposed monocytes produced more cytokines; however, mRNA expression did not increase in any cytokine genes. Therefore, the increases in cytokine production that we observed are likely due to enhanced translation.

How do circulating monocytes contribute to insulin resistance in peripheral (insulin-sensitive) tissues? As shown in this study, stimulated monocytes produce higher levels of cytokines (IL-1β, IL-6, MCP-1, and TNFα) that can travel in the circulation and interfere with the insulin signaling pathway in peripheral tissues [28]. Monocytes also can sense metabolic stress and differentiate into macrophages with high inflammation potential and perpetuate a chronic low-grade inflammatory state [29]. In line with this notion, TLR4 KO mice exhibit significantly less macrophage accumulation in fat [8] and macrophage-specific JNK1/2 double KO mice have inhibited M1 polarization [29] when fed a high-fat diet.

Another key finding in this study is that lipid infusion markedly enhanced monocyte secretion of IL-1β following LPS stimulation. In addition to the well described increases in plasma FFA, recent data from various groups, including ours, have shown that obese and type 2 diabetic subjects have elevations in plasma LPS concentration [30, 31], likely as a result of a dysfunctional intestinal barrier. In these individuals, FFA and LPS could act synergistically to enhance monocyte immune response leading to chronic systemic inflammation. This priming effect of FFA may be due to the upregulation of TLR4 level, as we have observed *in vivo* in this study and *in vitro* previously [20].

Obese and diabetic subjects have a high prevalence of atherosclerotic vascular disease and monocytes/macrophages play an important role in the etiology of atherosclerosis [32]. Activated monocytes are recruited to the intima and subintima, where they differentiate to inflammatory macrophages and promote formation of the atherosclerotic lesion [32]. There are substantial data linking TLR4 with atherosclerosis pathogenesis. For example, coronary artery disease is associated with increased monocyte TLR4 expression and an inflammatory phenotype [33, 34]. Also, TLR4 polymorphisms with attenuated receptor signaling are associated with decreased risk of atherosclerosis [35]. Therefore, TLR4 activation by FFA could stimulate monocyte recruitment to blood vessels and promote atherosclerosis.

In summary, our study demonstrates that in human subjects, a small increase in plasma FFA upregulates TLR4 expression and stimulates downstream pathways in circulating monocytes. We conclude that the activation of these pathways in monocytes could be involved in the low-grade inflammatory state seen with obesity and type 2 diabetes. We also conclude that targeting TLR4 may be an effective way to decrease inflammation and improve glucose metabolism in these individuals.

## Supporting information

S1 Dataset All individual data. (XLSX)

S1 Fig Flow cytometry gating strategy for identification of monocytes from PBMC. (TIF)

S2 Fig Effect of lipid infusion on plasma LPS concentration. (TIF)

S1 Table Effect of lipid/saline infusion on metabolic profile. (TIF)
