# Supplementary figures and images for: A low dose lipid infusion is sufficient to induce insulin resistance and a pro-inflammatory response in human subjects
Source: PLoS One. 2018 Apr 12;13(4):e0195810. doi: 10.1371/journal.pone.0195810 (PMC5897027; doi:10.1371/journal.pone.0195810)

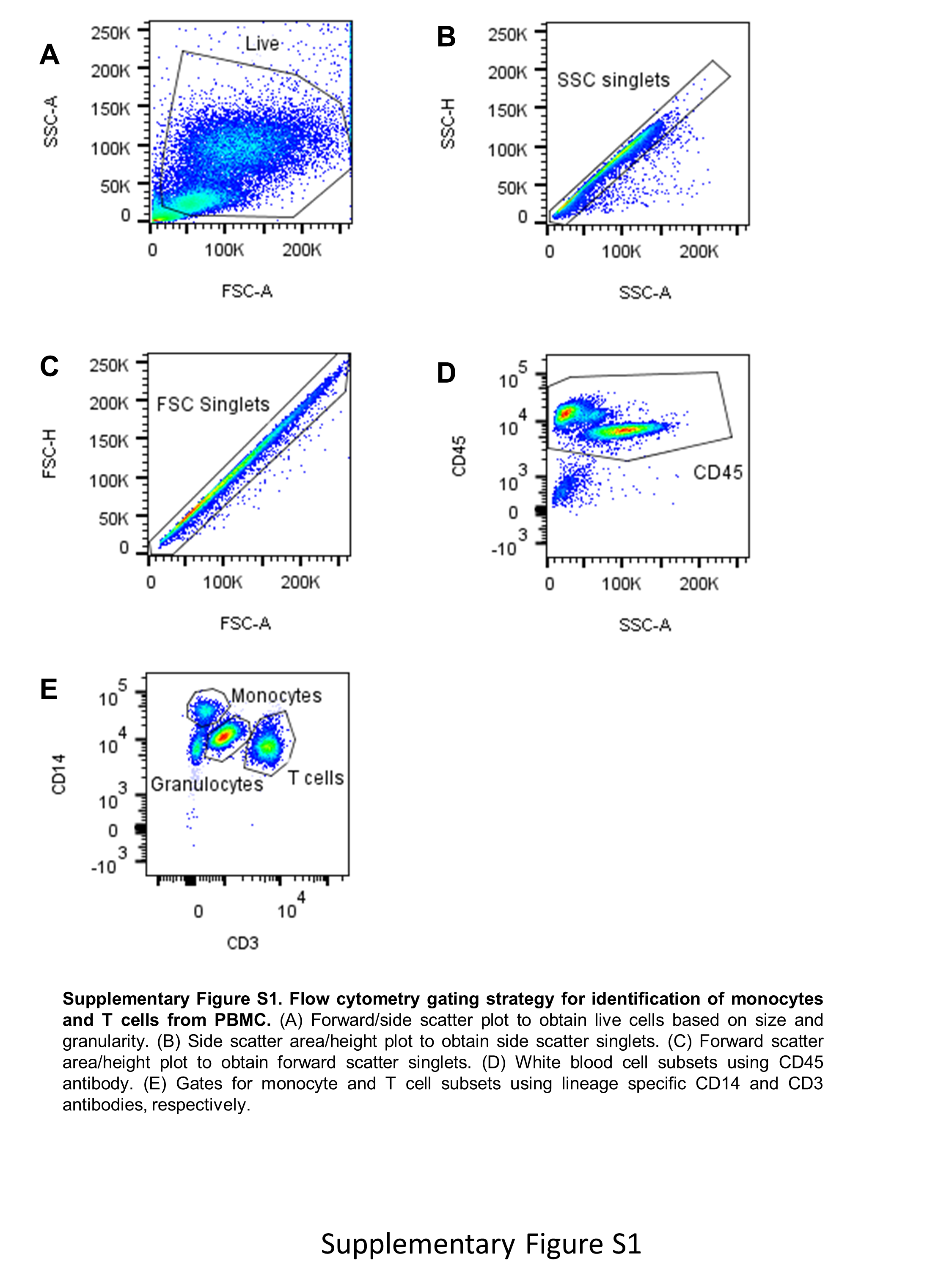

Supplement: S1 Fig — (TIF) [file pone.0195810.s002.TIF]

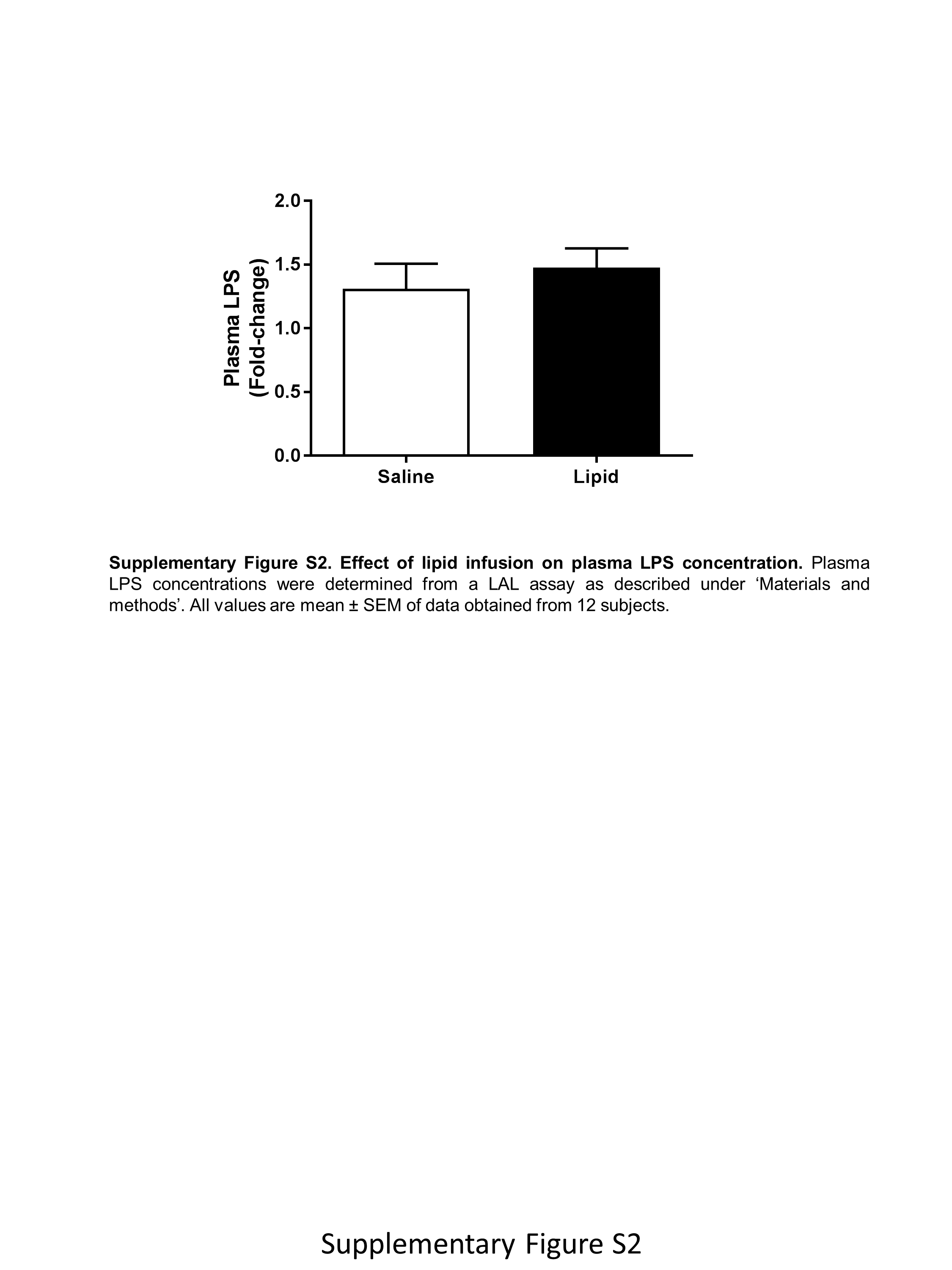

Supplement: S2 Fig — (TIF) [file pone.0195810.s003.TIF]

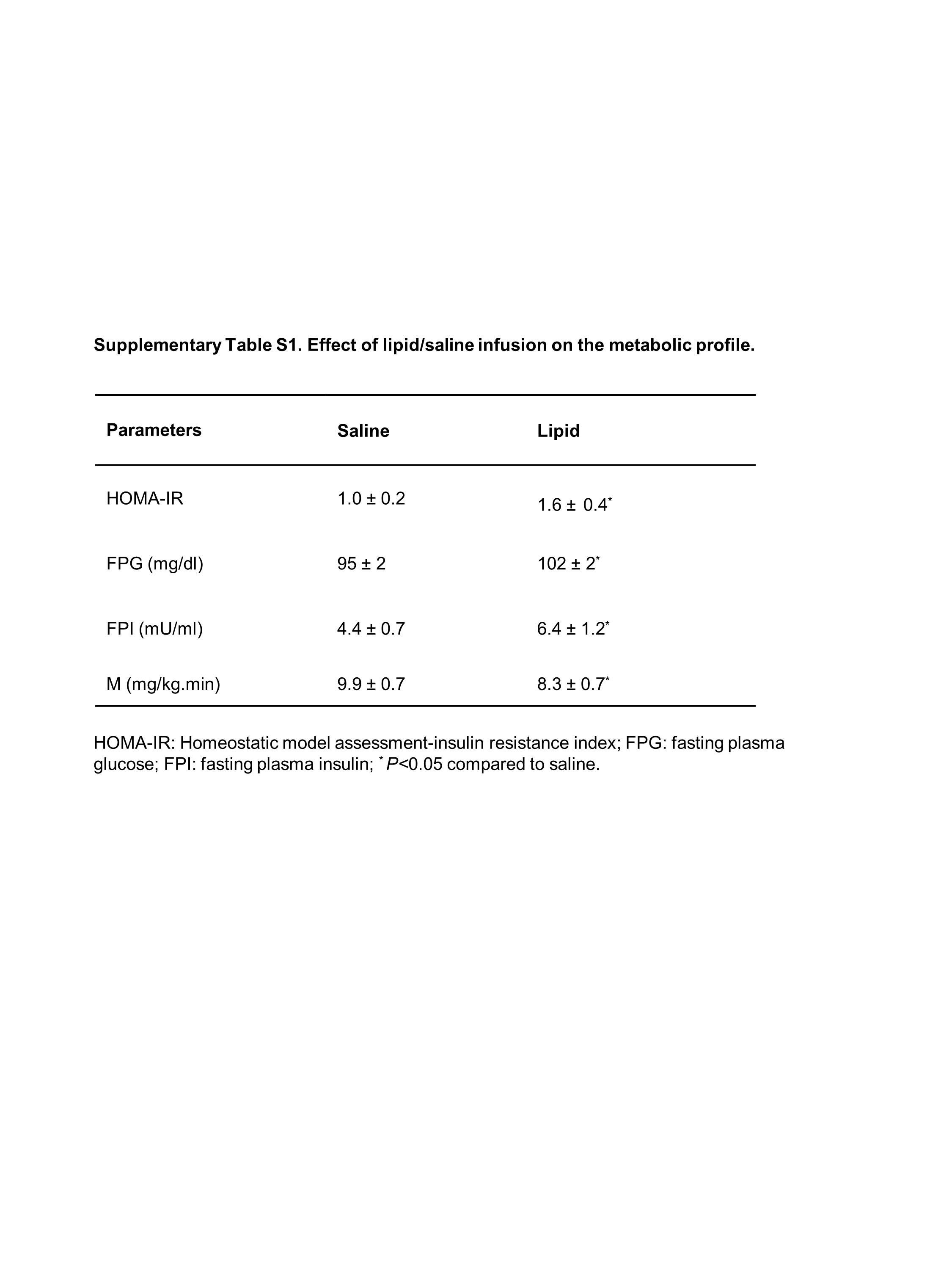

Supplement: S1 Table — (TIF) [file pone.0195810.s004.TIF]
